# Heat‐induced maternal effects shape avian eggshell traits and embryo development and phenotype at high incubation temperatures

**DOI:** 10.1002/ece3.10546

**Published:** 2023-09-22

**Authors:** Alexander J. Hoffman, Leslie Dees, Haruka Wada

**Affiliations:** ^1^ Department of Biological Sciences Auburn University Auburn Alabama USA; ^2^ Auburn High School Auburn Alabama USA

**Keywords:** avian eggshell, embryo physiology, hormesis, maternal effects, phenotypic plasticity, thermal stress

## Abstract

Phenotypic plasticity is an important avenue by which organisms may persist in the face of rapid environmental change. Environmental cues experienced by the mother can also influence the phenotype of offspring, a form of plasticity called maternal effects. Maternal effects can adaptively prepare offspring for the environmental conditions they will likely experience; however, their ability to buffer offspring against environmental stressors as embryos is understudied. Using captive zebra finches, we performed a maternal‐offspring environmental match‐mismatch experiment utilizing a 2 × 2 × 2 factorial design. Mothers were exposed to a mild heat conditioning (38°C) or control (22°C) treatment as juveniles, an acute high heat (42°C) or control (22°C) treatment as adults, then paired for breeding. The eggs produced by those females were incubated at a hyperthermic (38.5°C) or optimal temperature (37.2°C). We found that when mothers were exposed to a mild heat conditioning as juveniles, their embryos exhibited reduced water loss, longer development times, and produced hatchlings with heavier pectoralis muscles when incubated at high incubation temperatures, compared to embryos from control mothers. Mothers exposed to both the mild heat conditioning as juveniles and a high heat stressor as adults produced eggs with a higher density of shell pores and embryos with lower heart rates during development. However, there was a cost when there was a mismatch between maternal and embryo environment. Embryos from these conditioned and heat‐stressed mothers had reduced survival at control incubation temperatures, indicating the importance of offspring environment when interpreting potential adaptive effects.

## Graphical abstract

Mothers can prepare their offspring for future conditions through maternal effects. Here, we demonstrate that zebra finch mothers exposed to a mild heat conditioning as juveniles produce embryos that are more resistant to water loss and truncated development times at high incubation temperatures. Mothers exposed to both the mild heat conditioning and then a high heat stressor as adults produced eggs with a higher density of shell pores and embryos with lower heart rates during development and heavier pectoralis muscles at hatch when incubated at a high temperature.

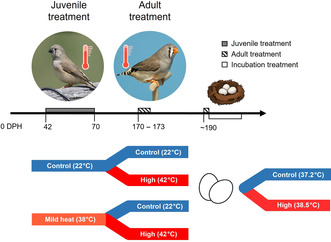

## 1 INTRODUCTION

Thermal biology drives geographical range limits of a species, and those that cannot rapidly adapt or migrate may experience severely diminished populations or extinction in the face of climate change (Thomas et al., 2006). Populations with slower growth rates, such as those with long generation times, risk extinction before genetic adaptation to the new environment can occur (Chevin et al., 2010). However, phenotypic plasticity (the ability of one genotype to produce multiple phenotypes) may give organisms a faster mechanism of persistence in rapidly changing environmental conditions compared to genetic adaptation. In one form of phenotypic plasticity, the environmental conditions experienced by parents can also affect the phenotype of offspring without alteration of DNA. Such plasticity is known as intergenerational phenotypic plasticity, or maternal effects, when mediated by the mother (Marshall & Uller, 2007). Maternal effects were once thought of as deviations from normal offspring development, but have been recognized for their importance in influencing offspring phenotype and evolutionary processes (Bernardo, 1996). Maternal effects can improve the fitness of offspring in a changing environment by adjusting their phenotype to match the expected environmental conditions (Mousseau & Fox, 1996). These adjustments are often called anticipatory maternal effects (Marshall & Uller, 2007). However, maternal effects may not always be beneficial to offspring fitness. In some cases, maternal effects may increase maternal lifetime fitness while having a detrimental effect on the performance or survival of current offspring (Bernardo, 1996; Marshall & Uller, 2007). These are known as selfish maternal effects (Bernardo, 1996; Marshall & Uller, 2007). This is in large part because selection acts to maximize fitness of the mother rather than that of the individual offspring, although they are often positively correlated (Bernardo, 1996). Thus, in order for maternal effects to increase offspring fitness, the environment experienced by the mother must be heterogeneous and an accurate predictor of the environment that will be experienced by offspring.

The strength and direction of maternal effects can be determined in part by the life history and resources available to the mother. Stressful environments often induce maternal effects at a cost to the mother, imposing a trade‐off between self‐maintenance and the current reproductive bout (Marshall & Uller, 2007; Sheriff & Love, 2013). Thus, maternal effects that increase offspring fitness are more likely to occur when the plasticity is not costly to the mother, such as when the environmental stressor is mild enough or temporally far enough away from reproduction to not incur maternal trade‐offs (Berrigan & Scheiner, 2004). Mothers may also avoid facing trade‐offs if they have a tolerance to the stressful conditions via phenotypic plasticity of their own, such as through conditioning hormesis (Calabrese et al., 2007; Costantini & Metcalfe, 2010). In such scenarios, mild stressor exposure adjusts the phenotype of an organism, promoting an increased tolerance to a higher severity of the stressor later on in life, (Calabrese et al., 2007; Costantini & Metcalfe, 2010). Maternal within‐generation plasticity may result in offspring with phenotypes better matched to their environments, as mothers with phenotypes that shift due to environmental cues may provide more accurate predictors of future conditions (Kuijper & Hoyle, 2015; Leimar & McNamara, 2015). Therefore, a mother's capacity to promote maternal effects can interact with, and in some cases may be dependent upon, the plasticity of their own phenotype. For example, *Drosophila melanogaster* exposed to variable thermal conditions during early ontogeny had better thermal tolerance than those exposed to high or constant temperatures and also produced offspring with increased thermal performance at constant temperatures, as measured by locomotor performance (Cavieres et al., 2019). However, experiments that incorporate multiple stressor exposure points experienced by mothers are limited, though such data may be critical in determining how within‐generation plasticity influences maternal effects and the stress tolerance of the next generation.

Ambient temperature is an ecologically variable environmental factor that is a strong driver of maternal effects in many species, influencing offspring traits such as dispersal, reproduction, and growth (Burgess & Marshall, 2011; Donelson et al., 2016; Salinas & Munch, 2012). Maternal exposure to increased temperatures has also been demonstrated to induce anticipatory maternal effects specifically, shaping offspring phenotypes to optimize their fitness in the ambient temperatures they experience postnatally (Burgess & Marshall, 2011; Crill et al., 1996; Salinas & Munch, 2012; Schiffer et al., 2013; Zizzari & Ellers, 2014). For example, threespine stickleback offspring had larger body sizes when reared at the same temperature at which their mothers had previously acclimated to (Burgess & Marshall, 2011). However, the bulk of the published research on temperature‐induced maternal effects has focused on invertebrates and ectothermic vertebrates and examined offspring performance postnatally (Shama et al., 2014). As such, the potential for maternal effects to improve embryonic development and survival has been understudied, especially in endotherms.

Embryos of many species have not yet developed the physiological systems necessary to detect environmental cues and adaptively adjust their phenotype (Uller, 2008). Potentially, maternal effects can circumvent this issue by producing offspring pre‐conditioned to the otherwise detrimental thermal conditions. Although avian embryos develop outside of the mother in an egg, providing a virtually closed environment, prenatal maternal effects play an important role in many species (von Engelhardt & Groothuis, 2011). Embryos of avian species are generally able to withstand low ambient temperatures for relatively prolonged periods, as parents must leave the nest to forage, leading to temporary cooling of the egg (Conway & Martin, 2000; DuRant et al., 2013; Mueller et al., 2015; Webb, 1987). In contrast, most embryos cannot withstand hyperthermic conditions for very long (Mueller et al., 2015; Webb, 1987). Rising incubation temperatures increases embryonic metabolic demands, resulting in higher oxygen consumption, water loss, and CO_2_ build‐up, eventually leading to desiccation, respiratory or cardiac failure, and death (Mortola, 2009). A high eggshell gas conductance can ameliorate some of these effects by increasing the rate at which oxygen diffuses in and CO_2_ diffuses out. However, this high gas conductance comes with the trade‐off of increasing water loss to potentially fatal levels (Mortola, 2009). The rate of gas exchange necessary to meet embryonic metabolic demands is influenced by the pore density and thickness of the eggshell, and therefore, these traits can largely impact development, survival, and the environment *in ovo*. Eggshell pore density and thickness differs across species ranges due to adaptation to varying environmental conditions such as temperature, humidity, and altitude, which is thought to occur across many generations (Morales et al., 2013; Rahn et al., 1977). However, if these eggshell characteristics display a degree of plasticity and could be adjusted due to the thermal conditions experienced by the mother, it could in part benefit their offspring when faced with rapid changes in ambient temperature in a single generation.

**FIGURE 1:**
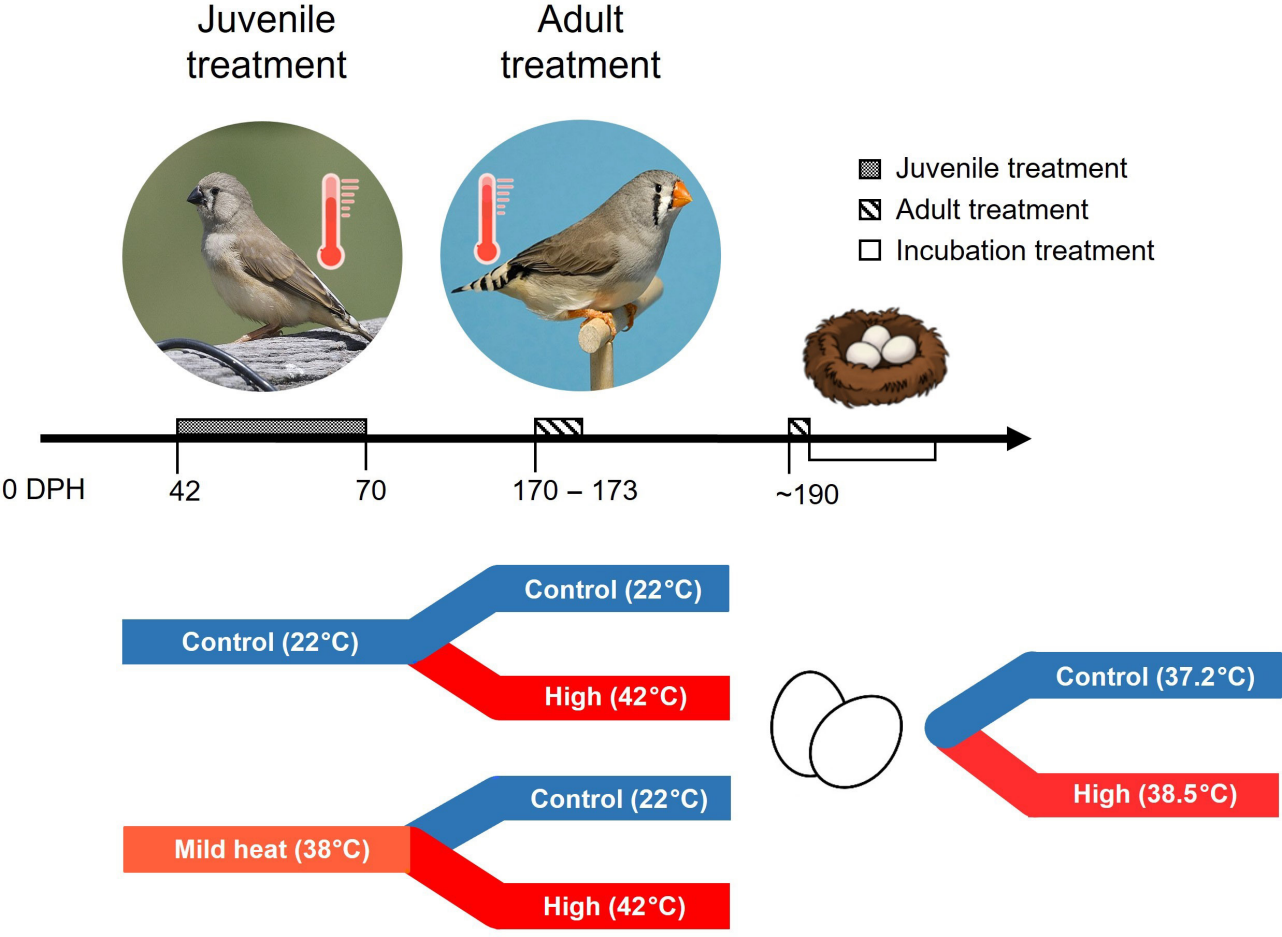
Schematic representation of the experimental timeline and depiction of treatment groups. Beginning at 42–45 days post‐hatch (DPH), females were exposed to either a mild heat (38°C) or control (22°C) treatment over a 28‐day period. In adulthood, starting at 170–173 DPH, these females were then exposed to either a high heat stressor (42°C) or control temperature (22°C) over a 3‐day period in a 2 × 2 factorial design. Several weeks later, the eggs produced by these females were removed from the nest and artificially incubated at an optimal (37.2°C) or high (38.5°C) incubation temperature.

In this study, we used a captive colony of zebra finches (*Taeniopygia castanotis*) to investigate whether maternal exposure to heat induces maternal effects in their offspring's phenotype and whether those changes are mediated through eggshell characteristics. Using a 2 × 2 × 2 factorial design, we examined how the maternal thermal environment influences embryo physiology, development, and survival at optimal and high incubation temperatures. To test this, we exposed juvenile females to a mild heat conditioning (38°C) or control (22°C) temperature treatment every other day for 28 days (Figure 1). This conditioning protocol can be considered a mild heat stressor because the temperature of 38°C is just above the upper critical temperature of the thermoneutral zone for the species (between 34.9 and 37.5°C) as determined by Wojciechowski et al. (2021), but has also been shown to not increase blood oxidative damage (Costantini et al., 2012). In the wild, even some of the hottest areas in Australia (such as near Alice Springs and Telfer) historically experience less than 28 days a month with temperatures above 35°C in the summer (Australian Government Bureau of Meteorology, 2022). However, the number of days per month with such high temperatures is increasing and is predicted to increase even further as a result of climate change (Conradie et al., 2020). As adults, the finches were then exposed to either a high heat stressor (42°C) or control temperature (22°C) treatment for 3 consecutive days (Figure 1). These females were paired to breed with non‐experimental males, and the resulting eggs laid were incubated for the duration of embryonic development at either a control temperature (37.2°C), or a high temperature (38.5°C), the latter of which has been shown to decrease hatching success in this species (Wada et al., 2015). We then measured several developmental and physiological parameters of offspring such as embryonic heart rate and development time, change in egg mass during incubation, and hatchling morphology. We also measured eggshell pore density and thickness to determine whether these characteristics can be influenced by the thermal conditions experienced by the mother, and whether this plasticity is associated with changes in offspring phenotype. We hypothesized that exposure to a mild heat conditioning treatment as juveniles would induce an anticipatory maternal effect, resulting in mothers producing offspring that are better suited to cope with high incubation temperatures, regardless of the thermal conditions mothers were exposed to as adults prior to breeding. Specifically, we predicted that these embryos would exhibit less water loss, lower heart rates, higher organ masses, and eggshells with increased pore density when compared to embryos from juvenile control mothers. We also predicted that non‐conditioned mothers exposed to an acute and high‐intensity thermal stressor in adulthood prior to breeding would induce a selfish maternal effect, producing offspring that have reduced body and organ masses and survival regardless of incubation temperature, and increased heart rates and water loss at high incubation temperatures.

## 2 MATERIALS AND METHODS

### 2.1 Animal diet and husbandry

Experimental females used in this experiment were obtained from our captive colony of zebra finches (*Taeniopygia castanotis*) at Auburn University. The details of the husbandry and experimental treatment of the mothers used in this study have been previously published (Hoffman et al., 2018). Briefly, female nestlings from 26 breeding pairs were used in this study. The finches received ad libitum mixed seeds (Kaytee Supreme (finch), Chilton, WI), water, grit, and cuttlefish bone as well as a once‐weekly supplement of vitamin drops and spinach and daily supplement of egg mixture (hard‐boiled chicken egg, white bread, and cornmeal). Upon reaching 10 days post‐hatch (DPH), nestlings were temporarily removed from the nest to be weighed and metal ID bands with a unique four‐digit number were attached to their leg. Juveniles were separated from their parents at 39–43 DPH and kept with other juveniles until the start of treatment.

### 2.2 Juvenile treatment

A total of 48 females underwent the juvenile conditioning treatment, following a protocol that has been previously established (Costantini et al., 2012) and used in our laboratory (Hoffman et al., 2018). The oldest offspring in each nest was randomly assigned to either the control or mild heat treatment group, after which we alternated treatment group assignments while accounting for sex in order to balance treatments within each nest. At 42–45 DPH, half of the juvenile females began the mild heat treatment and were exposed to a temperature of 38°C every other day, 3 h per day, for 28 days in a brooder (TLC‐50; Brinsea Products Inc.) as previously described (Costantini et al., 2012; Hoffman et al., 2018). For this species, body temperature begins to increase at ambient temperatures higher than approximately 35.9°C, leading to mild hyperthermia (Wojciechowski et al., 2021). This increase in body temperature limits water loss by creating a thermal gradient allowing for better heat dissipation and may initiate stress responses such as glucocorticoid secretion and heat shock protein synthesis (Feder & Hofmann, 1999; Wingfield et al., 1998). The other half of the juvenile females were used as a control group and were placed into brooders in the same manner as the mild heat group, but instead set to our general aviary room temperature of 21–23°C. Although this temperature is below the species thermoneutral zone, it was used as a control treatment because it is the temperature at which they, as well as previous generations, were reared. When undergoing treatment bouts, we placed 3–7 finches in brooders beginning between 8:30 and 9:30 a.m. local time. While in the brooders, finches received ad libitum cold water that was replaced at the first‐ and second‐hour mark of the 3‐h treatment period. When not in the brooder undergoing treatment, all birds were housed in general aviary temperature conditions (21–23°C) and kept in single‐sex tower cages (1–3 birds per cage) and received ad libitum of the same diet described above. Following the end of the 28‐day juvenile treatment period, birds were housed in these same conditions until the start of the adult‐stage treatment at 170–173 DPH.

### 2.3 Adult treatment

At 170–173 DPH, half of the females from each juvenile‐stage treatment were exposed to a high heat stress treatment of 42°C for 3 h a day, for 3 consecutive days as previously described (Costantini et al., 2012; Hoffman et al., 2018). This temperature is well above the upper critical temperature of the thermoneutral zone and has been shown to cause blood oxidative damage in this species (Costantini et al., 2012; Wojciechowski et al., 2021). The remaining females from each of the juvenile‐stage treatment groups served as the adult‐stage control group and were exposed to room temperature (21–23°C) in the same manner as above. This 2 × 2 factorial design resulted in four maternal treatment groups that were the different combinations of juvenile and adult treatment (Control–Control [CC]: *n =* 13, Control‐High [CH]: *n =* 14, Mild‐Control [MC]: *n =* 11, and Mild‐High [MH]: *n =* 9).

### 2.4 Female pairing

Females were paired with non‐experimental males and on the day of pairing, females first underwent a single additional 3‐h bout of their respective adult‐stage treatment (control or high heat) as described above. Pairs were housed in the same breeding conditions and received the same diet as previously described. A total of 43 females (*n*: CC – 12, CH – 12, MC – 10, MH – 9) successfully produced eggs that underwent the incubation stage treatment.

### 2.5 Egg collection and incubation treatment

After pairing, nests were checked for eggs daily at approximately 10:00 a.m. local time. Once laid, eggs were removed from the nest and marked with a unique ID number using a fine‐tip permanent marker. The first egg laid in the clutch was replaced with a false egg made of modeling clay (Crayola Model Magic [white], Easton, PA) after removal, while any subsequent eggs removed were not replaced. The first egg laid was randomly assigned to an incubation treatment (a control or high incubation temperature) and following that the first laid egg of each subsequent nest was assigned a treatment group in an alternating manner to balance treatment allocation among the first eggs of the clutch. After the first egg, treatment group assignments alternated within each clutch to balance laying order and nest of origin between incubation temperature treatments. Therefore, in general, there were multiple embryos in both treatment groups for each female.

Newly laid eggs were transported to the lab where they were weighed and then placed into one of two incubator treatments, representing a control or high incubation temperature. Those assigned to the control group (*n* = 125 embryos) were transferred into an incubator (Brinsea Octagon 20 Advance EX incubator) set to 37.2°C for the entirety of embryonic development. This temperature was chosen as it is very close to the average incubation temperature of captive zebra finches (Zann & Rossetto, 1991) and was found to be optimal for the species when considering both hatching success and post‐hatch survival (Wada et al., 2015). Individuals assigned to the high incubation temperature (*n* = 106 embryos) were placed into an incubator set to a temperature of 38.5°C, which is slightly above the range of average incubation temperatures for wild and laboratory‐kept zebra finches (Zann & Rossetto, 1991), for the entire incubation period. In this species, an incubation temperature of 38.4°C can result in lower hatching success overall and in lower overall and lean body mass in male offspring during their postnatal growth period (Wada et al., 2015, 2018) and therefore considered outside the thermal optimum. Both incubators were set to maintain a relative humidity of 55%. Incubators were calibrated to the desired temperature using a reference thermometer placed at level with the eggs. Approximately three times per week, incubators were taken off of their rockers and allowed to sit level for at least 1 h before reading the thermometer to determine temperature accuracy. The average measured temperature in control and high‐temperature incubators were 37.13°C ± 0.51 and 38.45°C ± 0.42 (mean ± SD), respectively. No incubator replicates were used; however, both incubators were distanced only ~20 cm apart and kept in a temperature‐controlled room with no foot traffic.

### 2.6 Heart rate measurements

Embryonic heart rates were measured using the Buddy® digital heart rate monitor (Avian Biotech, Vetronic Services, Abbotskerswell). The Buddy® digital heart rate monitor uses a non‐invasive method which quantifies heart rate by the amount of infrared light absorbed by the pulsing embryonic blood. The Buddy® monitor has been used in a variety of studies on oviparous embryos, often examining the effects of incubation temperature on reptilian embryos (Du et al., 2009; Hall & Warner, 2018; Hulbert et al., 2017). More specifically, the Buddy® monitor has also been used in studies where poultry and turkey embryos were exposed to non‐optimal temperatures, as well as studies on other avian species using a variety of stimuli (Sheldon et al., 2018). More recently, a thorough investigation using the Buddy® system has been done on wild zebra finch embryos (Sheldon et al., 2018).

Heart rate measurements were recorded repeatedly at two different time points during an individual's embryonic development, first at embryonic day (ED) 4, which is at ~28.9% of development for embryos in the control temperature incubator and ~29.4% of development in the high‐temperature incubator. The second measure was taken at ED 11 for embryos in the control incubator and at ED 10 for embryos in the high incubator, which is ~79.6% and ~73.5% of development, respectively. Heart rate data were collected between 2:00 and 5:00 p.m. local time to account for any circadian variation.

When measuring heart rates, eggs were individually removed and placed horizontally (pointed tip facing digital display) on the measuring platform of the monitor before closing the lid. A timer was started as soon as the egg was removed from the incubator, and the amount of time(s) that the embryo had been out of the incubator at each heart rate (bpm) measurement was recorded. This value was used in the analysis to account for the decrease in heart rate when eggs were removed from the incubator and began to cool. Heart rates were recorded every 10 s after the first recording until the egg had been outside of the incubator for ~70 s. If reliable heart rates could not be picked up by the monitor by the time the egg had been out of the incubator for ~40 s, the egg was flipped 180° on the platform. Measurements were considered reliable when the output values were consistent and continuous, with the “heart icon” on the digital display blinking. The eggs did not remain inside the monitor for longer than ~90 s in total. The heart rate measurement taken between the 50 and 60 s mark was used in statistical analyses. For day 4 measurements, eggs were immediately placed back into their respective incubator after heart rates had been recorded. For day 10/11 measurements, each egg was weighed after recording the heart rate to calculate mass loss before being returned to incubators. Eggs did not remain outside of the incubators for longer than ~240 s total while measuring heart rate and egg mass. The order in which eggs were removed from the incubator for measurements was also recorded to be taken into account in the statistical analysis. When no heart rate signal could be found using the monitor, eggs were candled using a flashlight to determine infertility or mortality. If an egg was found to have a crack due to handling or collisions inside the incubator, the heart rate was not measured and that individual was not used in survival analysis. When an embryo was found to be dead and had no cracks in the shell, the developmental stage (very early, early, mid, late, very late, pipping) at which mortality occurred was estimated and recorded for later use.

### 2.7 Organ measurements

All incubators were checked a minimum of three times daily for potential hatchlings. When a hatchling was found, it was removed from the incubator, weighed, and then euthanized via isoflurane inhalation as per approved protocol. Dissected tissues included the residual yolk sac, heart, and pectoralis muscle, and the tissues were weighed prior to freezing. After removal, all dissected tissues were then flash frozen in liquid nitrogen before being placed in a −80°C freezer, while the carcass was placed in a freezer at −20°C for later use.

### 2.8 Eggshell measurements

Pore density was determined using a protocol refined for songbird eggs by Stein & Badyaev (2011) with some modification. Briefly, three pieces of shell from each egg were chosen representing the blunt end, equator, and pointed end of the egg. These pieces were then placed in a biopsy case and incubated in hot NaOH bath for 3 min to remove the eggshell membrane. Following this, the shells were rinsed in ddH2O, then dried in an incubator set to 41°C. Once dry, the thickness of the shell was measured using a micrometer (Mitutoyo, cat number 395‐371‐30, 0.001 mm resolution) to the nearest 0.001 mm. Shells were then incubated in 2.5% nitric acid for 12 s, followed by a rinse in ddH_2_O. The shells were dried once again in an incubator set to 41°C. Once dry, the number of eggshell pores was counted using a Nikon Eclipse microscope. One researcher measured the thickness, and another researcher counted all the pores for all samples. Both researchers were blind to the treatment groups from which the eggshells came.

### 2.9 Statistical analyses

Statistical analysis was done using RStudio Team (2020) utilizing the packages “nlme” and “survival” and graphs were constructed using the “ggplot2” and “survminer” packages. Statistical tables were constructed using the “tab_model” function in the package “sjPlot.” Statistical significance was determined as *p* ≤ .05, and all models included the maternal ID (“FID” or “nestid”) as a random effect to account for multiple eggs originating from the same mother. To examine the effects of our experimental treatments on dependent variables we first ran initial “full” models that included the juvenile*adult*incubator treatment interaction term and the lower‐order two‐way interaction terms, as well as predictors that were believed likely to have a large effect size or plausible biological effect on some aspects of the model. Predictors remained in the model regardless of significance, while non‐significant interaction terms were removed when lower‐order conditional main effects, lower‐order interaction terms, and other predictors were also non‐significant. Assumptions of linearity of the data were tested by plotting the residuals versus fitted values. Assumptions of normality of residuals were tested via histogram plots of the data for linear models. For linear models, regression coefficients were used for estimates of mean change in response variables, while odds ratios were used for estimates in generalized linear regression models. Estimates of uncertainty were represented via 95% confidence intervals, calculated using the functions “intervals” and “confint” for linear regressions and generalized linear mixed‐effects models, respectively.

#### 2.9.1 Egg mass and heart rate

Linear mixed‐effects models (LMM) were used to analyze effects on all egg mass, heart rate, and hatchling mass variables. To test the effect of maternal treatment on initial egg mass (mass on the day egg was laid and prior to incubation treatment), we used a model with juvenile treatment, and adult treatment as fixed factors, lay order (the sequential order in which the egg was laid for each female) as a covariate, as well as a maternal juvenile*adult treatment interaction term. Incubation treatment was not included in this egg mass analysis, as the initial egg mass was taken before being placed in the incubator. The change in egg mass over the first ~77% of embryonic incubation was analyzed using a model with juvenile, adult, and incubation treatment as fixed factors, a juvenile*incubation treatment interaction term as well as the embryonic heart rates at both ~30% and ~77% of embryonic development included as covariates. The effect of maternal and incubation treatments on embryonic heart rates at ~30% development was analyzed using juvenile, adult, and incubation treatment as fixed factors, as well as the two‐way juvenile*adult interaction term. The effect of maternal and incubation treatments on embryonic heart rates at ~77% development was analyzed using juvenile, adult, and incubation treatment as fixed factors, as well as the two‐ and three‐way interaction terms, and initial egg mass as a covariate. A further post hoc analysis was done using an LMM with a categorical variable “fulltreatment” representing the eight combinations of juvenile, adult, and incubation treatments as a fixed factor, and initial egg mass as a covariate. The coded variable represents the eight distinct treatment groups resulting from the 2 × 2 × 2 factorial design and allows for post hoc pairwise comparisons.

#### 2.9.2 Embryonic development time and hatchling organ mass

The effect of maternal juvenile and incubation treatments on embryonic development time was analyzed using an LMM with juvenile, adult, and incubation treatment as fixed factors, a juvenile*incubation treatment interaction term, as well as the embryonic heart rate at ~77% of embryonic development included as a covariate. A further post hoc analysis was done using a LMM which included a categorical treatment variable representing the four combinations of maternal juvenile and incubation treatment as a fixed factor, adult treatment as a fixed factor, as well as the embryonic heart rate at ~77% of embryonic development included as a covariate. Pectoralis mass was analyzed using a LMM with juvenile, adult, and incubation treatment as fixed factors, a juvenile*incubation treatment interaction term, hatchling body mass, and incubation time as covariates. A further post hoc analysis was done using a LMM which included the juvenile*incubation categorical treatment variable (see above) as a fixed factor, adult treatment as a fixed factor, and hatchling body mass and incubation time as covariates. The effect of maternal and incubation treatments on body (total hatchling mass prior to euthanasia) mass of hatchlings was analyzed using a LMM which included juvenile, adult, and incubation treatment as fixed effects, their two‐ and three‐way interaction terms, initial egg mass, incubation time, and embryonic heart rate at ~77% of embryonic development as covariates. The model examining the effects of treatments on hatchling residual yolk (the mass of yolk internalized from egg stores that were present at hatch) and heart mass included juvenile, adult, and incubation treatment, their two‐ and three‐way interaction terms, as well as hatchling total body mass as a covariate.

#### 2.9.3 Eggshell characteristics

We used LMMs for all models to determine the effect of maternal and incubation treatments on eggshell characteristics. The difference in eggshell pore density between embryos that lived and died was analyzed using a model which included survival (whether the embryo died during development or successfully hatched) as a factor, initial egg mass as a covariate, and their two‐way interaction term. The difference in eggshell thickness between embryos that lived and died was analyzed using a model which included survival as a factor, initial egg mass as a covariate, and their two‐way interaction term. We analyzed the effect of maternal treatment on eggshell pore density using a model which included juvenile and adult treatment as fixed factors, as well as the two‐way interaction term, and egg mass as a covariate. A further post hoc analysis was done using a LMM which included a juvenile*adult categorical treatment variable (see above) as a fixed factor, and egg mass as a covariate. We analyzed the effect of maternal treatment on eggshell thickness using a model which included juvenile and adult treatment as fixed factors, as well as their two‐way interaction term, and egg mass as a covariate. A further post hoc analysis was done using a LMM which included a juvenile*adult categorical treatment variable as a fixed factor, and egg mass as a covariate.

#### 2.9.4 Embryo survival

The effect of incubation treatment on embryonic survival was analyzed using a generalized linear mixed‐effects model (GLMM) which included juvenile, adult, and incubation treatment as fixed effects, as well as the two‐ and three‐way interaction terms. The data was then subset by incubation treatment to examine the effects of maternal treatments within different incubation temperatures using GLMs which included the maternal juvenile*adult categorical treatment variable as a fixed factor. Kaplan–Meier curves were generated using models which included the maternal juvenile*adult categorical treatment variable as a fixed factor.

## 3 RESULTS AND DISCUSSION

**FIGURE 2:**
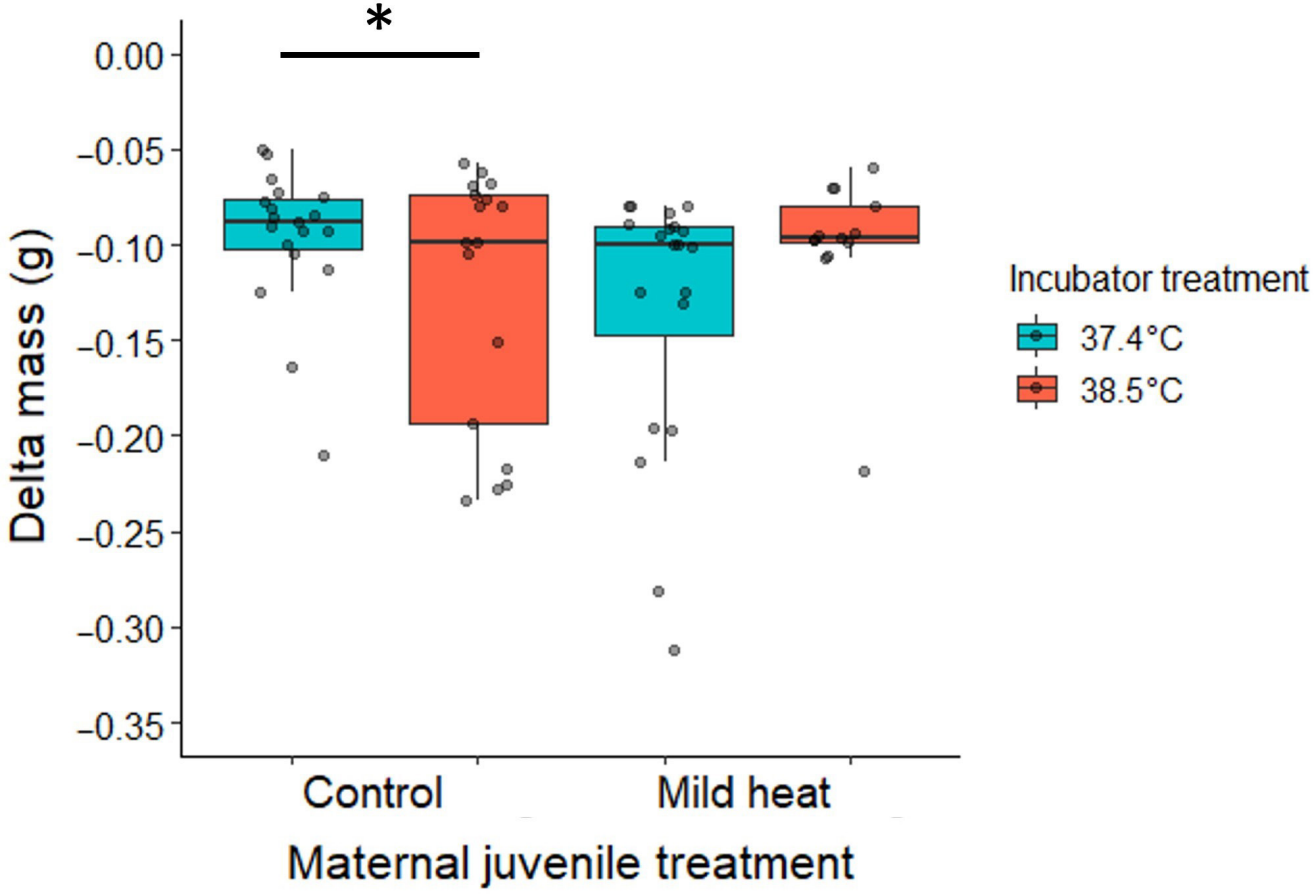
At high incubation temperatures, embryos showed no increase in egg mass loss when produced by mothers exposed to a mild heat conditioning as juveniles, compared to embryos at control incubation temperatures (significance indicated as follows for all graphs *p ≤ .05, **p ≤ .01, ***p ≤ .001). The change in egg mass (g) occurring from the day the egg was laid until approximately 77% of the incubation period. The *x*‐axis denotes the treatment mothers received as juveniles, either a control (22°C) or mild heat (38°C) temperature for a prolonged period. The legend denotes the different incubation treatment groups that embryos produced by those mothers were exposed to, either a control (37.2°C) or high incubation temperature (38.5°C). Boxplots show the median (horizontal line), lower quartile (median to end of box), upper quartile (median to top of box), minimums and maximums within 1.5 × interquartile range (whiskers), and outliers (data points beyond the whiskers).

For avian embryos, the demand for oxygen generally increases as temperatures rise, leading to an increased production of CO_2_ as well as higher water loss through diffusion across the eggshell (Mortola, 2009). While the ratio of CO_2_ lost to O_2_ gained is between 0.70 and 0.75 in the avian egg, the O_2_ molecule also has 27% lower mass, meaning that the mass exchanged due to the diffusion of these molecules is nearly equivalent (Mortola, 2009). Therefore, during the course of incubation, the change in mass of the embryo can be attributed almost entirely to water loss. In our experiment, we used the change in mass as an indirect measure of water loss. When we compared the water loss among eggs produced by control mothers, we saw an expected pattern. Embryos from juvenile control mothers lost 5.63 × 10^−2^ g (±0.047, ±95% CI) more water at a higher incubation temperature compared to embryos incubated at the control incubation temperature (Figure 2) (Linear mixed model [LMM]: *t* = −2.50, df = 22, *p* = .0204, random effect (SD) = 1.14 × 10^−6^). However, mothers who were exposed to the mild heat conditioning as juveniles produced embryos that were seemingly more resistant to water loss at high ambient temperatures. Embryos from these heat‐conditioned mothers lost similar amounts of water at higher incubation temperatures compared to embryos from heat‐conditioned mothers incubated at a control temperature (Figure 2) (LMM: *t* = 0.429, df = 22, *p* = .672, random effect (SD) = 1.14 × 10^−6^). In contrast, the thermal treatment mothers received as adults approximately 3 weeks before breeding had no effect on the water loss during incubation. This effect of maternal treatment on water loss cannot be attributed to differential egg size alone, as there were no differences in initial egg mass among the groups (Table S1).

**FIGURE 3:**
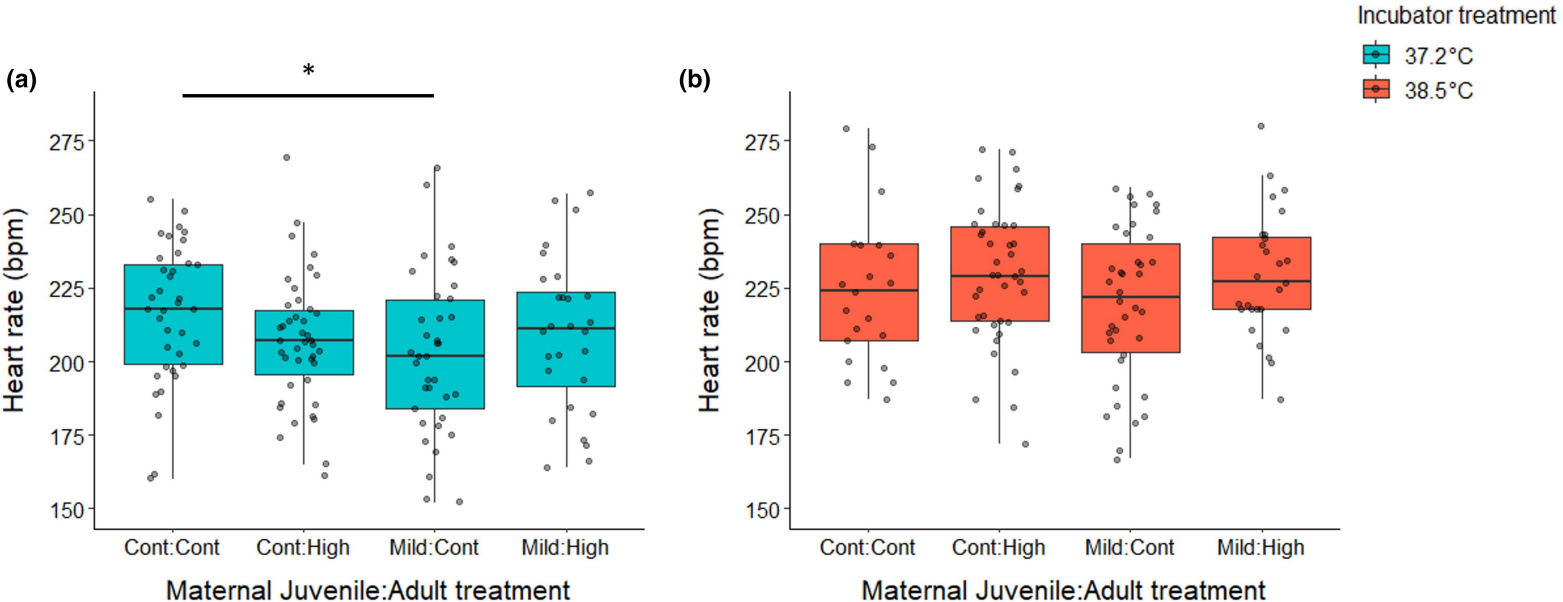
Embryos produced by mothers exposed to the mild heat conditioning as juveniles had lower heart rates, but only at the control incubation temperature. (a) The heart rates of embryos in the control incubation treatment at approximately 30% of the development period. (b) The heart rates of embryos in the high incubation treatment at approximately 30% of the development period. For each group on the *x*‐axis, the first word denotes the treatment the mothers received as juveniles (control or mild heat) and the second word denotes which treatment they received as adults (control or high heat). The legend denotes the different incubation treatment groups that embryos produced by those mothers were exposed to, either a control (37.2°C) or high incubation temperature (38.5°C). Boxplots show the median (horizontal line), lower quartile (median to end of box), upper quartile (median to top of box), minimums and maximums within 1.5 × interquartile range (whiskers), and outliers (data points beyond the whiskers).

**FIGURE 4:**
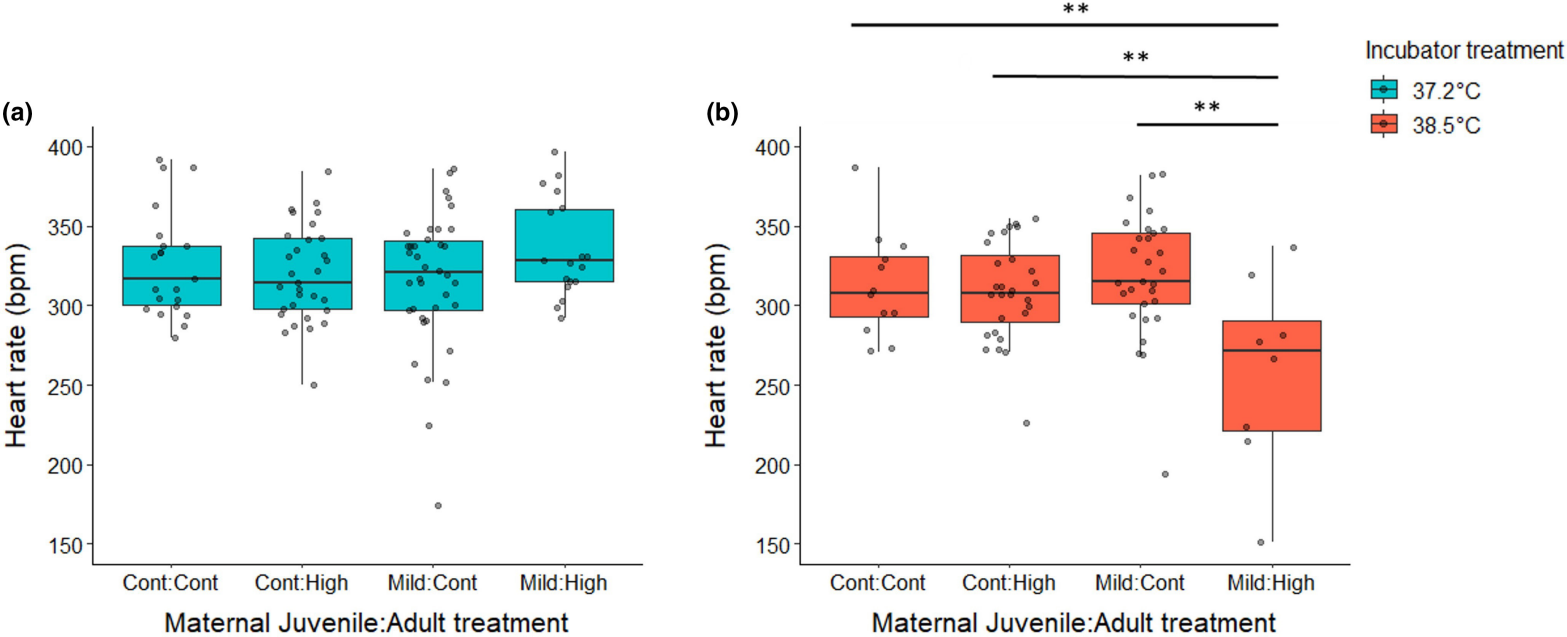
Embryos produced by mothers exposed to the mild heat conditioning as juveniles and high heat stressor as adults had lower heart rates, but only at the high incubation temperature. (a) The heart rates of embryos in the control incubation treatment at approximately 77% of the development period. (b) The heart rates of embryos in the high incubation treatment at approximately 77% of the development period. For each group on the *x*‐axis, the first word denotes the treatment the mothers received as juveniles (control or mild heat) and the second word denotes which treatment they received as adults (control or high heat). The legend denotes the different incubation treatment groups that embryos produced by those mothers were exposed to, either a control (37.2°C) or high incubation temperature (38.5°C). Boxplots show the median (horizontal line), lower quartile (median to end of box), upper quartile (median to top of box), minimums and maximums within 1.5× interquartile range (whiskers), and outliers (data points beyond the whiskers).

At standardized humidity, differences in embryonic water loss can be attributed in part to metabolic rate. Therefore, we investigated how maternal heat exposure shapes the metabolic rate of offspring as embryos. To do this, we measured embryonic heart rate – which is frequently used as a proxy for metabolic rate in birds and reptiles due to its strong correlation with oxygen consumption (Du et al., 2009; Sheldon et al., 2018) – at ~30% and ~77% of embryonic development. We found that exposure to high incubation temperatures increased heart rates of embryos at ~30% of development across all samples, but there was no difference seen due to the mother's treatment at this temperature (Table S1). However, at the control incubation temperature, embryos from mothers that experienced the mild heat as juveniles (Mild‐Control) had heart rates 13.1 bpm (±11.4, ±95% CI) lower than those produced by mothers with no prior heat exposure (Control–Control) (Figure 3a) (LMM: *t* = −2.28, df = 223, *p* = .0237, random effect (SD) = 1.58). Later on in embryonic development (at ~77%), we again observed a maternal effect on heart rate. When incubated at the high temperature, embryos from mothers exposed to both heat treatments (Mild‐High) had significantly lower heart rates than embryos from all other maternal treatment groups (Figure 4b) (Table S2 – LMM: df = 142 all *p* < .01, random effect (SD) = 4.98). Reduced heart rates could act as an adaptive maternal effect, especially at high incubation temperatures. As heart rates increase with ambient temperature, so does the risk of cardiac arrhythmia and arrest (Mueller et al., 2022). Zebra finch embryos already exhibit exponential increases in heart rate and oxygen consumption late in development, which can make them potentially more susceptible to death as a result of hyperthermic conditions at this time (Pearson et al., 1999; Vleck et al., 1979). Therefore, lower heart rates as exhibited by embryos from the Mild‐High mothers may increase the chance for survival at high temperatures. As expected, when all samples were analyzed together there was a negative correlation between the embryonic heart rate at ~77% of embryonic development and change in egg mass during development. For each 1 beat per minute (bpm) increase in heart rate, we saw a 4.76 × 10^−4^ g (±3.31 × 10^−4^, ±95% CI) increase in mass (water) loss (LMM: *t* = −2.92, df = 35, *p* = .006, random effect (SD) = 1.15 × 10^−6^). However, this pattern did not hold true for all treatment groups. At control incubation temperatures, embryos from mothers exposed to both heat treatments (Mild‐High) exhibited the aforementioned increase in water loss as heart rates increase (LMM: *t* = −2.56, df = 11, *p* = .0264, random effect (SD) = 1.85 × 10^−6^). However, at high incubation temperatures, embryos from this same group showed no significant increases in water loss as heart rates increased (LMM: *t* = −1.04, df = 6, *p* = .339, random effect (SD) = 3.22 × 10^−6^). While these decreases in water loss and heart rate at high incubation temperatures exhibited by embryos from heat‐conditioned mothers may represent an adaptive change in physiology, reducing the risk of death via desiccation or cardiac failure, such physiological changes may have trade‐offs with other components of embryonic development.

**FIGURE 5:**
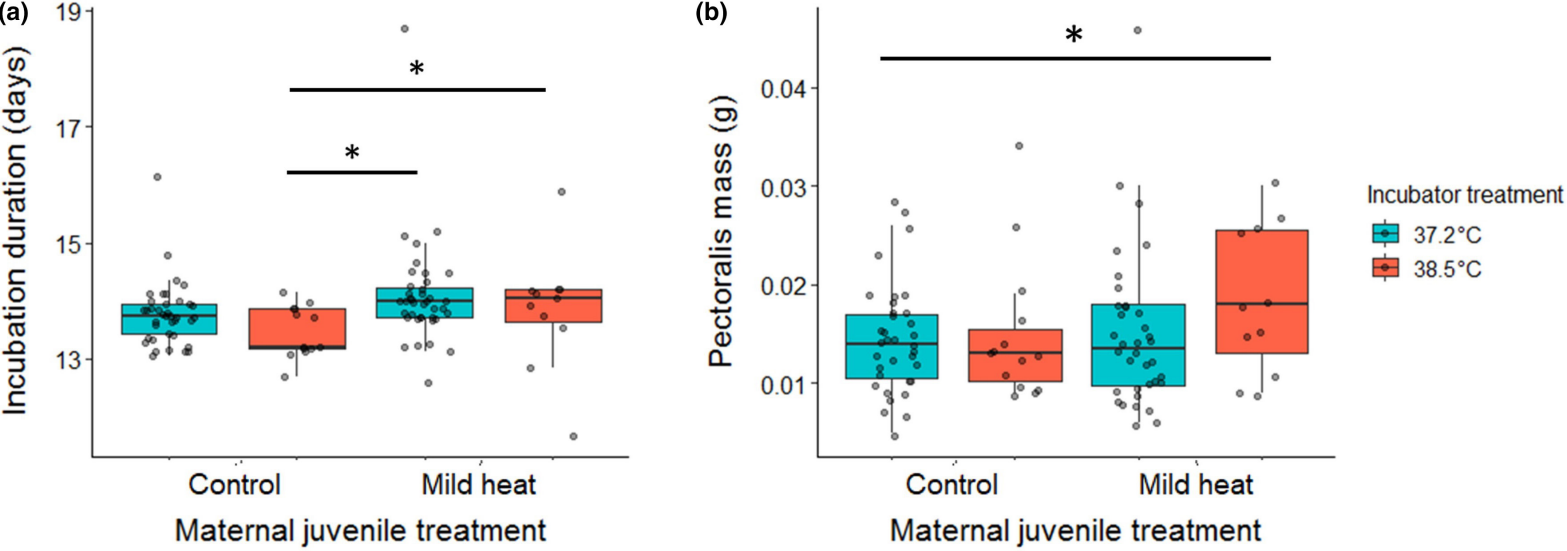
Embryos produced by mothers exposed to mild heat as juveniles had longer development durations and heavier pectoralis muscles at hatch. (a) The total incubation duration (days) of embryos developed at different temperatures. The *x*‐axis denotes the treatment mothers received as juveniles, either a control (22°C) or mild heat (38°C) temperature for a prolonged period. The legend denotes the different incubation treatment groups that embryos produced by those mothers were exposed to, either a control (37.2°C) or high incubation temperature (38.5°C). (b) The total weight (g) of offspring pectoralis muscles after hatching. The *x*‐axis denotes the treatment mothers received as juveniles, either a control (22°C) or mild heat (38°C) temperature for a prolonged period. The legend denotes the different incubation treatment groups that embryos produced by those mothers were exposed to, either a control (37.2°C) or high incubation temperature (38.5°C). Boxplots show the median (horizontal line), lower quartile (median to end of box), upper quartile (median to top of box), minimums and maximums within 1.5× interquartile range (whiskers), and outliers (data points beyond the whiskers).

Non‐optimal incubation temperature can result in changes of embryonic development time and/or differential hatchling morphology (Gurley et al., 2018; Rubin et al., 2021; Wada et al., 2015, 2018). For many avian species including the zebra finch, increases in egg incubation temperature result in decreased incubation periods (Wada et al., 2015). In such situations, embryos may prioritize development of essential organs such as the brain and heart at the expense of others (Mortola, 2009). Here, maternal juvenile treatment increased embryonic development time; however, this effect was dependent on the incubation temperature the embryos were exposed to Table S3. As expected for embryos produced by juvenile control mothers, those that were incubated at the high temperature had a development time 0.439 days (±0.450, ±95% CI) shorter than those incubated at the control temperature (Figure 5a) (LMM: *t* = −1.96, df = 47, *p* = .056, random effect (SD) = 0.719). In contrast, embryos produced by mothers exposed to the mild heat conditioning as juveniles had development times 0.974 days (±0.815, ±95% CI) longer than embryos from juvenile control mothers when incubated at high temperatures (Figure 5a) (LMM: MH‐CH: *t* = 2.40, df = 47, *p* = .0202, random effect (SD) = 0.719). These relatively longer development times demonstrated by embryos from conditioned mothers, even at high incubation temperature, were similar to development times of embryos incubated at the optimal temperature (Figure 5a) (CC‐MH: *β* = 0.535, *t* = 1.45, df = 47, *p* = .154, random effect (SD) = 0.719). As expected, there was a significant negative correlation between development time and embryonic heart rate at ~77% of embryonic development, a pattern similar to that demonstrated in zebra finches and other species (Amos & Tazawa, 1999; Hiroshi, 2005; Sheldon & Griffith, 2018). More specifically, we also saw that the longer development times at high incubation temperatures exhibited by embryos produced by heat‐conditioned mothers were correlated with their reduced heart rates at ~77% of embryonic development (LMM: *t* = −2.35, df = 25, *p* = .027).

In some scenarios, slow growth of avian embryos is associated with increased individual quality that affects lifetime fitness (Ricklefs et al., 2017). However, there are potential trade‐offs as longer development times also increase mortality risks such as by adverse weather events or predation (Ricklefs et al., 2017). While slower embryonic development utilizes more overall energy, the rate of energy consumption is lower (Rahn & Ar, 1974; Ricklefs et al., 2017). This reduced energy consumption rate can explain why the longer development times exhibited by embryos from heat‐conditioned mothers may be an adaptive maternal effect at high incubation temperatures. Late in avian embryonic development, metabolic demands are at their highest and the risk of death due to hypoxia, water loss, and CO_2_ build‐up is increased (Mortola, 2009). Therefore, avoiding further increases in metabolic demands with increases in temperature during this time could lower mortality risks. Longer development times may also have beneficial implications for fitness by reducing damage at a cellular level. For example, in some avian species there is a negative correlation between embryonic development time and damage (lipid peroxidation and DNA breakage) suggesting a trade‐off between embryonic development rate and oxidative damage, most likely mediated by metabolic rate (Tsunekage, 2015; Tsunekage & Ricklefs, 2015). It has been suggested that oxidative stress is a key mediator of life history trade‐offs by influencing traits such as reproduction and survival, and so avoiding such oxidative insults as embryos by increasing development time may increase lifetime fitness (Costantini, 2008; Metcalfe & Alonso‐Alvarez, 2010; Monaghan et al., 2009).

Interestingly, the differential developmental patterns observed between treatment groups were also associated with morphology differences at hatching. While there was no effect of maternal treatment on hatchling body, heart, or residual yolk mass, we observed that hatchlings produced by mothers who were exposed to the mild heat conditioning as juveniles had heavier pectoralis muscles when incubated at a high temperature (Table S3). These hatchlings had pectoralis muscles 5.03 × 10^−3^ g (±4.91 × 10^−3^, ±95% CI) heavier than hatchlings produced by juvenile control mothers incubated at a control temperature (Figure 5b) (LMM: pectoralis: *t* = 2.05, df = 61, *p* = .0450, random effect (SD) = 5.94 × 10^−7^). The pectoralis muscles can act as an invaluable thermoregulatory organ in birds, as they are the most essential site for shivering thermogenesis which allows birds to maintain body temperature at ambient temperatures below their thermoneutral zone (Cooper, 2002; Dawson & O'Connor, 1996; Hohtola, 2004; Marsh & Dawson, 1989). Thus, while increased pectoralis muscle mass is associated with adaptations to low ambient temperatures, it is interesting that in this study we saw higher masses in response to increased temperatures experienced by mothers and embryos. However, the heavier pectoralis muscles in hatchlings may still represent an adaptive change. In chickens, increased incubation temperatures have been demonstrated to result in higher hatchling pectoralis mass, as well as a greater thermotolerance later in life (Collin et al., 2007; Piestun et al., 2013a, 2013b). Larger pectoralis muscles may also give individual birds an advantage in poor conditions, outside of thermoregulatory effects. An increase in pectoralis muscle without an increase in total body mass (as seen in this study) can improve flight performance and efficiency in birds (Verspoor et al., 2007), which could be adaptive in poor conditions, potentially by increasing predator avoidance capabilities or allowing increased foraging activity levels when food availability is low or unpredictable (Bautista et al., 1998; Lima, 1993; Noakes et al., 2020; Witter et al., 1994). Furthermore, if offspring are forewarned of poor post‐hatch conditions via the environment experienced by their mothers and their own pre‐natal environment, increased pectoralis muscles may be adaptive by providing protein reserves to persevere during times of food shortage (Bize et al., 2007). The increased pectoralis mass suggests a potential adaptive maternal effect, as the higher mass and longer development times at high incubation temperatures were not at the cost of reduced overall body, heart, or residual yolk mass (Table S4). However, we cannot rule out that this change in embryo phenotype could incur a cost that only becomes apparent later in life, such as reduced longevity or reproductive output in adulthood. Whether such trade‐offs occur may also depend on the thermal environment the offspring would experience post‐hatch. Therefore, future experiments examining potential long‐term costs or benefits of such maternal effects would do well to utilize multiple post‐hatch temperature regimes when evaluating offspring performance.

The underlying mechanisms through which maternal thermal experience influenced embryonic heart rates, development times, and pectoralis mass as seen in this experiment is unknown; however, one potential candidate is through maternal hormone deposition in the egg. Hormone‐mediated prenatal maternal effects have been well documented in avian species for their ability to influence development and signal environmental conditions to offspring (Groothuis et al., 2005). Moreso, the amount of steroid hormones a mother deposits in the egg yolk can be influenced by the environmental conditions she experiences (Groothuis et al., 2005). For example, laying hens exposed to a moderate heat challenge (30°C) for 5 weeks produced eggs with higher concentrations of testosterone, progesterone, and estradiol which influenced hatchling quality and growth (Bertin et al., 2013). Furthermore, increased exposure to steroid hormones *in ovo* has been demonstrated to alter the same embryo phenotypic traits measured in this study. For example, Rock pigeon (*Columba livia*) embryos with experimentally elevated yolk androgen levels exhibited higher heart rates during development and stimulated prenatal growth (Wang et al., 2023). With regard to incubation length, a study showed that zebra finch eggs injected with testosterone had longer embryonic development times than control eggs (Von Engelhardt et al., 2006). Increased embryonic steroid hormone exposure can also influence pectoralis mass, as European starlings (*Sturnus vulgaris*) exposed to experimentally elevated yolk corticosterone as embryos had heavier pectoralis muscles and improved flight performance as fledglings (Chin et al., 2009). Therefore, the alterations in embryonic heart rates, development times, and pectoralis mass from mothers exposed to the thermal treatments, even when incubated at high temperatures, may be the result of differences in circulating steroid hormones in mothers as a result of their prior thermal exposure.

Differential development patterns may not only be attributed to metabolic rate but also to eggshell traits. Lower pore density and thicker eggshells decrease gas conductance and therefore water loss. However, such morphology would also limit the rate of diffusion of oxygen and CO_2_ across the eggshell, potentially decreasing the chance of survival at high temperatures. Therefore, we examined the role eggshell traits played in embryonic survival. We found that successfully hatched individuals had 18.4 (±2.60, ±95% CI) pores per cm^2^ of eggshell compared to 16.1 (±1.50, ±95% CI) pores per cm^2^ of eggshell in embryos that died during development (LMM: *t* = 2.36, df = 75, *p* = .021, random effect (SD) = 2.83). However, no difference in eggshell thickness between embryos that lived and died was observed (LMM: *t* = 1.37, df = 79, *p* = .175, random effect (SD) = 0.0044).

While it is clear that eggshell characteristics can influence the physiology, development, and survival of avian embryos, little is known about their potential for plasticity and ability to facilitate anticipatory maternal effects. Therefore, we examined how eggshell pore density and thickness are influenced by thermal conditions experienced by the mother. We found that among mothers exposed to the high heat treatment as adults, those that also underwent the mild heat conditioning treatment as juveniles produced eggs with 4.26 (±3.96, ±95% CI) more pores/cm^2^ compared to those that did not (LMM: MH‐CH: *t* = 2.20, df = 29, *p* = .036, random effect (SD) = 2.38). We also saw that these mothers exposed to the heat treatments at both timepoints (Mild‐High) produced eggs with 5.38 (±4.36, ±95% CI) more pores/cm^2^ than Control–Control mothers as well (LMM: *t* = 2.52, df = 29, *p* = .018, random effect (SD) = 2.38), while we saw no differences in eggshell thickness due to experimental treatments (Table S5). Interestingly, this increase in pore density did not correlate with increased water loss, even at high incubation temperatures. This pattern may be due in part to the lower heart rates of embryos from mild‐high mothers at high temperatures, which paired with the increased number of pores potentially signals a reduced demand for oxygen rather than limitations in gas conductance.

**FIGURE 6:**
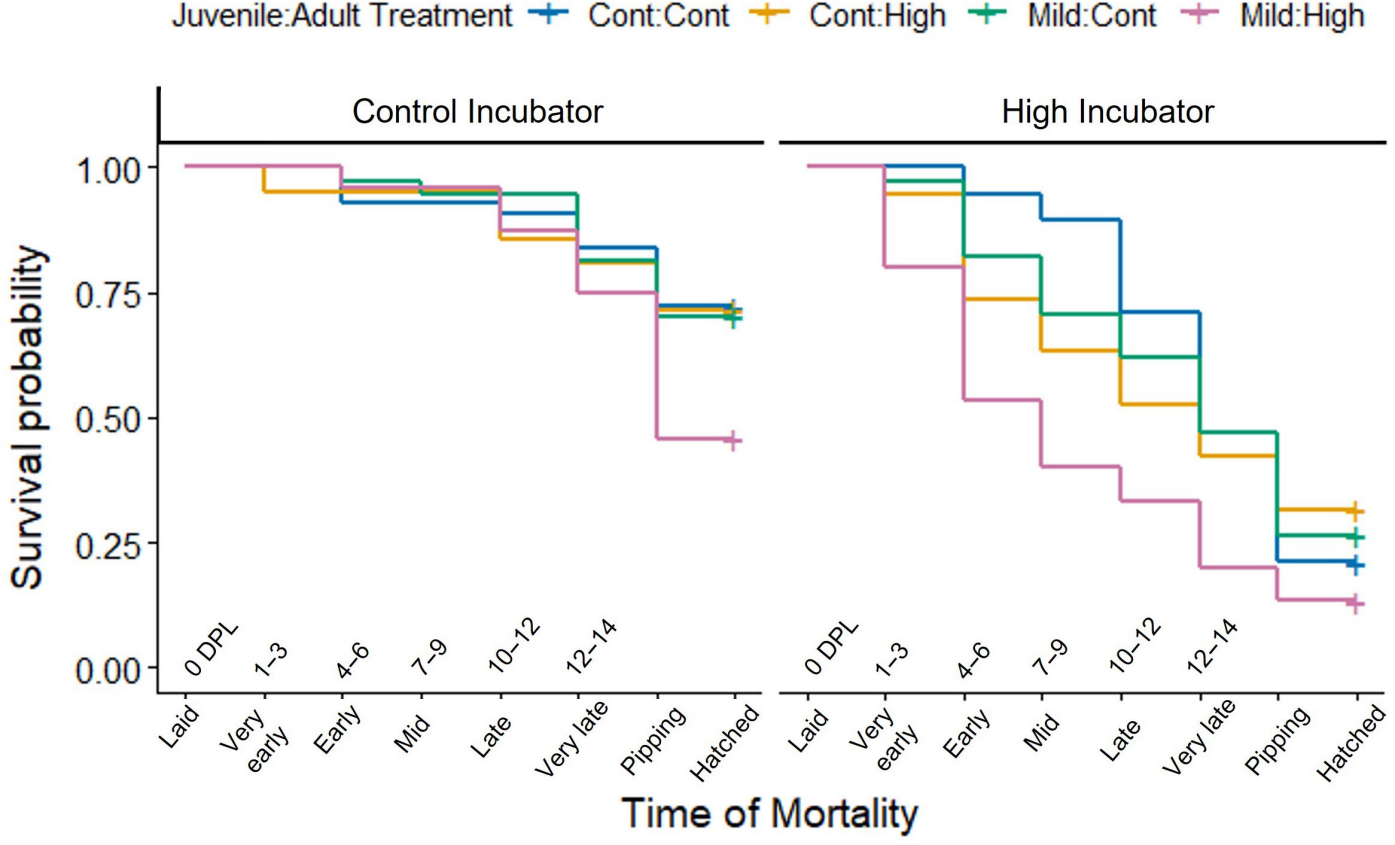
Embryonic survival in relation to maternal treatment at each incubation temperature. Survival probabilities of embryos at different points in embryonic development, grouped by the respective thermal treatments received by their mothers. The *x*‐axis denotes the estimated developmental stage (and the corresponding number of days) at which mortality of embryos occurred or if they successfully hatched, with “DPL” corresponding to “days post‐laid.” The left half of the graph shows embryonic survival at the control incubation temperature (37.2°C) while the right shows survival at the high incubation temperature (38.5°C). In the legend, the first word denotes the treatment the mothers received as juveniles (control or mild heat) and the second word denotes which treatment they received as adults (control or high heat).

In zebra finches, an incubation temperature of just one degree Celsius above optimum has been shown to decrease both hatching success and lean body mass in male embryos (Wada et al., 2015, 2018). As expected, here we saw that embryos incubated at the high temperature were 5.42 times more likely to die than those incubated at the control temperature (GLMM: *β* = 1.69, 95% CI = [0.384, 3.12], *z* = 2.45, *p* = .014, random effect (SD) = 0.245). We presumed that the lower water loss, reduced heart rates, and higher pore density shown by embryos produced by heat‐exposed mothers would promote increased survival at high temperatures; however, this was not the case. Among embryos exposed to the high incubation temperature, maternal treatment had no effect on survival (Figure 6; Table S6). There are a few possible reasons for this non‐significance. It may be that any maternal effects potentially beneficial for embryos were not strong enough to counteract the negative effects of the high incubation temperature on embryo survival. Another possibility is that the potential benefits or constraints of those physiological changes are only seen on a different scale of observation and/or do not present themselves until later on in life. Early life cellular level damage can have effects persisting into adulthood. For example, high incubation temperatures and faster embryonic development rates were shown to reduce the length of telomeric DNA sequences in Japanese quail (Stier et al., 2020). This reduction in telomere length persisted into adulthood, and such shortening is associated with reduced longevity in many species (Heidinger et al., 2012). Therefore, while effects such as longer development times may not improve short‐term survival, it may help avoid increases in damage at high incubation temperature and improve survival outcomes in adulthood.

While we found no effect of maternal treatment on embryo survival at a high incubation temperature, we did observe a maternal effect at control temperatures. At the control incubation temperature, embryos from mothers that experienced the mild heat as juveniles were 3.05 times more likely to die if the mothers were also exposed to the high heat stress as adults (Mild‐High) (Figure 6) (GLM: MC‐MH: *β* = 1.12, 95% CI = [0.084, 2.19], *z* = 2.10, *p* = .036). This difference is largely due to the drop in survival late in the incubation period around pipping, where the Mild‐High group had a 29.2% drop in overall survival, compared to a 11.6%, 10.8%, and 9.6% decrease for the other three groups (Mild‐Cont, Cont‐High, Cont‐Cont respectively). Such a detrimental effect on offspring fitness may be due to the mismatch between maternal and offspring environment. Potentially, maternal exposure to elevated ambient temperatures during both development and adulthood was a strong signal that her offspring would also experience increased ambient temperatures, strongly necessitating a phenotypic change to maintain fitness (Sheriff & Love, 2013). However, at control incubation temperatures, the embryo's environment did not match the maternal conditions which induced the phenotypic change, resulting in decreased survival (Marshall & Uller, 2007).

While interpreting the adaptive significance of maternal effects is complex due to their highly context‐dependent nature, our study demonstrates the importance of designing experiments that incorporate several different time points and magnitudes of stressor exposure. Many of the effects we observed were the result of maternal exposure to a mild and prolonged heat conditioning as juveniles and its effect on the phenotype of embryos that developed at the high incubation temperature. Such changes in offspring phenotype as the result of an interaction between “matching” maternal and offspring environment may signal the presence of anticipatory maternal effects, however, further research is required. Our findings support previous studies which have demonstrated that the juvenile period is an important ontogenetic window for inducing not only within‐generation plasticity but also intergenerational plasticity. While the plasticity of the mother's phenotype was not the focus of this study, the maternal treatment protocol utilized in this study has been previously demonstrated to induce within‐generation plasticity in zebra finches (Costantini et al., 2012). Specifically, the juvenile mild heat treatment induced conditioning hormesis, preventing finches from suffering increased blood plasma oxidative damage after exposure to the high heat stressor as adults. The ability to induce adaptive maternal effects may interact with or even depend on the mother's own ability to induce within‐generation plasticity. As such, the mild heat conditioning of mothers may also induce conditioning hormesis in the offspring, dubbed “transhormesis” by Costantini (2022). However, we hesitate to claim the presence of a transhormetic effect in this study, as we did not measure physiological costs and saw no beneficial effects of maternal treatment on survival at high temperatures. Nonetheless, the duration of the mild heat conditioning likely played a significant role in its ability to generate plastic responses, as the 28‐day treatment period represents a significant portion of the developmental period in zebra finches (~90 days from hatching to sexual maturity) (Zann, 1996). Similar effects have previously been demonstrated, as adult sheepshead minnows exposed to 24°C and 34°C for 30 days produced offspring with increased growth at the temperature their parents were subjected to Salinas and Munch (2012). However, this parental effect was not seen when the parents were exposed to the temperatures for 7 days rather than 30 days. Findings such as these support the idea that environmental cues of a longer duration are more likely to induce anticipatory maternal effects, as they present a strong indicator that there will be a high correlation between the maternal and offspring environment.

Interestingly, the high heat stressor treatment as adults did not induce a selfish maternal effect, or any effect independent of its interaction with the juvenile mild heat treatment. This lack of effect is surprising, as the time just prior to and during a reproductive bout is considered another critical window for inducing maternal effects (Donelson et al., 2018). Stressful environments can often create a trade‐off between self‐maintenance and the current reproductive bout for a mother resulting in selfish maternal effects (Marshall & Uller, 2007). However, we observed no apparent detrimental effects of the maternal high heat stressor (independent of its interaction with the juvenile treatment) on the offspring or maternal phenotype (Hoffman et al., 2018). Potentially, trade‐offs were reduced in this study via plentiful resources, or the stressor was not strong enough or close enough to the reproductive bout to elicit a cost. Mothers had ad lib access to seed and water except when undergoing experimental treatments, potentially allowing them to increase consumption to negate detrimental effects of the stressor. Often a longer environmental cue near reproduction will result in a stronger intergenerational effect (Donelson et al., 2018; Dupont et al., 2013; Ho & Burggren, 2012; Salinas & Munch, 2012). If the high heat stressor mothers experienced as adults was a longer duration or closer to the reproductive bout, we may have observed a more pronounced effect. Another possibility is that we did not capture the phenotypic costs because we did not measure the affected traits, or because they were not present at the timescale at which we measured. Potentially, the cost of this stressor exposure for mothers may only be seen at a cellular level (Costantini et al., 2012) or may only be seen later in life such as reduced longevity (Costantini et al., 2014). Similarly, we measured phenotypic traits in offspring only prenatally and at hatch, and an induced selfish maternal effect may not be apparent until later on in development. Traits have different capacities for plasticity and involvement in trade‐offs, which underpins the importance of measuring multiple traits and factoring in life history in future studies on maternal effects moving forward (Angilletta Jr. & Angilletta, 2009).

Our results suggest that thermal conditions experienced by the mother early on in life can induce maternal effects that alter the phenotype and development of embryonic offspring. Such plasticity could play a key role in species that live in regions experiencing temperatures close to their upper thermal tolerance, which are thought to be at greater risk of diminished populations as a result of climate change. Our results also indicate that thermal conditions experienced by the mother, even very early on in life, can elicit a phenotypic change in her offspring prenatally. Such information is critical, as the embryos of many avian species develop near their upper thermal critical limits and have very little thermoregulatory capabilities of their own (Mortola, 2009; Webb, 1987). Even though avian parents can regulate egg temperature, their methods to prevent hyperthermia when ambient temperatures rise above the optimum incubation temperature often come at a significant cost to themselves (Grant, 1982). As climate change causes increased temperatures worldwide as well as an increase in extreme temperature events, the importance of understanding the rescue capabilities of phenotypic plasticity for species grows (Chevin et al., 2010). Understanding physiological and morphological adjustments of embryos due to maternal effects could improve our ability to predict population responses to climate change and pinpoint species that will be most at risk. Future studies in maternal effects would do well to utilize multiple ecologically relevant stressor magnitudes and exposure time points, as well as measuring a suite of traits in offspring over the course of their life history in varying environments.

## AUTHOR CONTRIBUTIONS

**Alexander J. Hoffman:** Conceptualization (equal); data curation (equal); formal analysis (equal); investigation (lead); methodology (equal); project administration (equal); supervision (equal); validation (equal); visualization (equal); writing – original draft (lead); writing – review and editing (equal). **Leslie Dees:** Data curation (equal); investigation (equal); methodology (equal); validation (equal); visualization (equal). **Haruka Wada:** Conceptualization (equal); data curation (equal); formal analysis (equal); funding acquisition (lead); investigation (equal); methodology (equal); project administration (equal); resources (lead); supervision (equal); validation (equal); visualization (equal); writing – original draft (equal); writing – review and editing (equal).

## FUNDING INFORMATION

This research was supported by the National Science Foundation grant IOS‐1553657 as well as by the USDA National Institute of Food and Agriculture [Hatch project 1008441], both awarded to H. Wada.

## CONFLICT OF INTEREST STATEMENT

The authors declare no competing interests.

## Supporting information


Data S1

Data S2

Tables S1–S6

## Data Availability

All data used for analyses are available in Data S1 and S2 and stored in the Dryad digital data repository (doi: 10.5061/dryad.8sf7m0cvk).
