# Supplementary material for: Heat‐induced maternal effects shape avian eggshell traits and embryo development and phenotype at high incubation temperatures
Source: Ecol Evol. 2023 Sep 22;13(9):e10546. doi: 10.1002/ece3.10546 (PMC10515880; doi:10.1002/ece3.10546)
Supplement: Supplementary file 3 — Tables S1–S6 [file ECE3-13-e10546-s002.docx]

Supporting Information for

**Heat-induced maternal effects shapes avian eggshell traits and embryo development and phenotype in high incubation temperatures**

Authors: Alexander J. Hoffman, Leslie Dees, Haruka Wada

Correspondence to: ajh0077@auburn.edu

**This file includes:**

Supporting Table S1 – S6

**Other Supporting Information for this manuscript include the following:**

Data files

- “Embryo Data for Hoffman et al V6” (.csv file)
- “Eggshell Data for Hoffman et al V3” (.csv file)

**Supporting table S1**

**
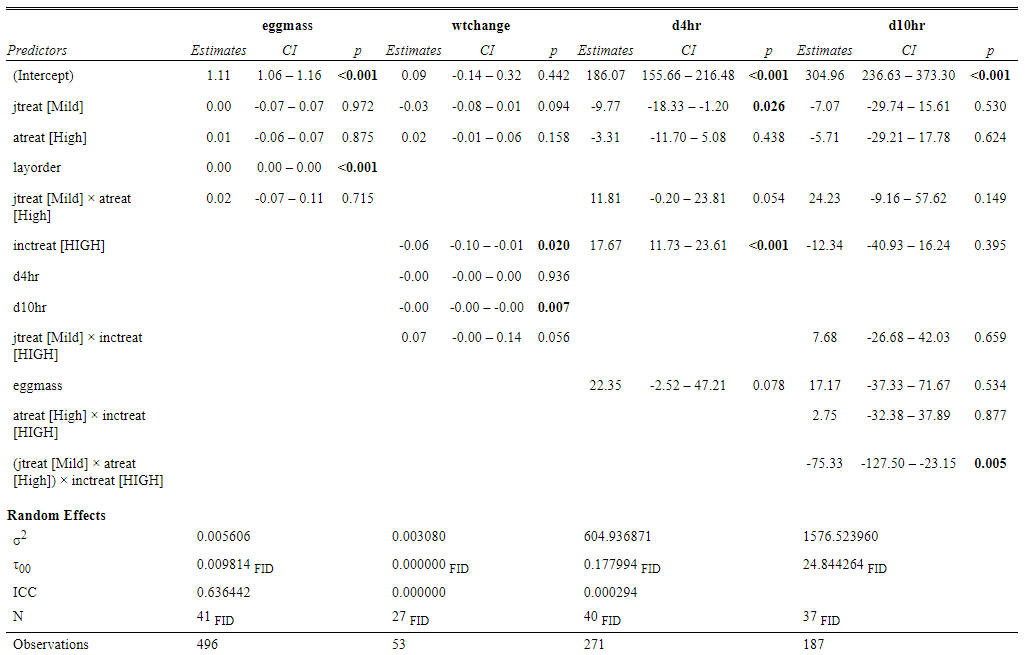
**

**Supporting Table S1. The statistical output of *LMMs* used to analyze the effect of maternal and incubation treatment on initial egg mass (g) (“eggmass”), egg mass change during incubation (g) (“wtchange”), early development heart rate (bpm) (“d4hr”), and late development heart rate (bpm) (“d10hr”).**

**Supporting table S2**

**
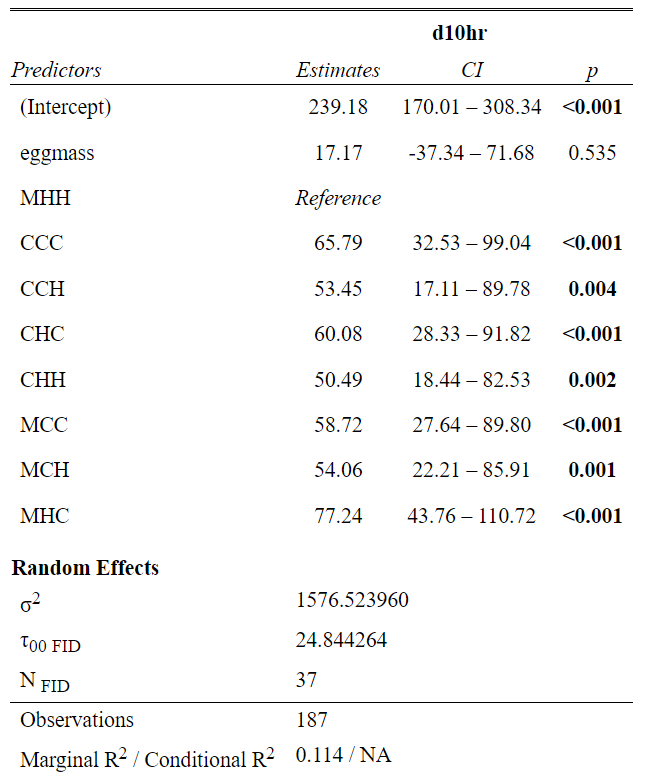
**

**Supporting Table S2. The statistical output of the *LMM* used to analyze the effect of maternal and incubation treatments on the heart rate of embryos late in development (bpm) (“d10hr”) using a categorical treatment variable that represents the eight combinations of maternal juvenile, maternal adult, and incubation treatments.**

**Supporting table S3**

**
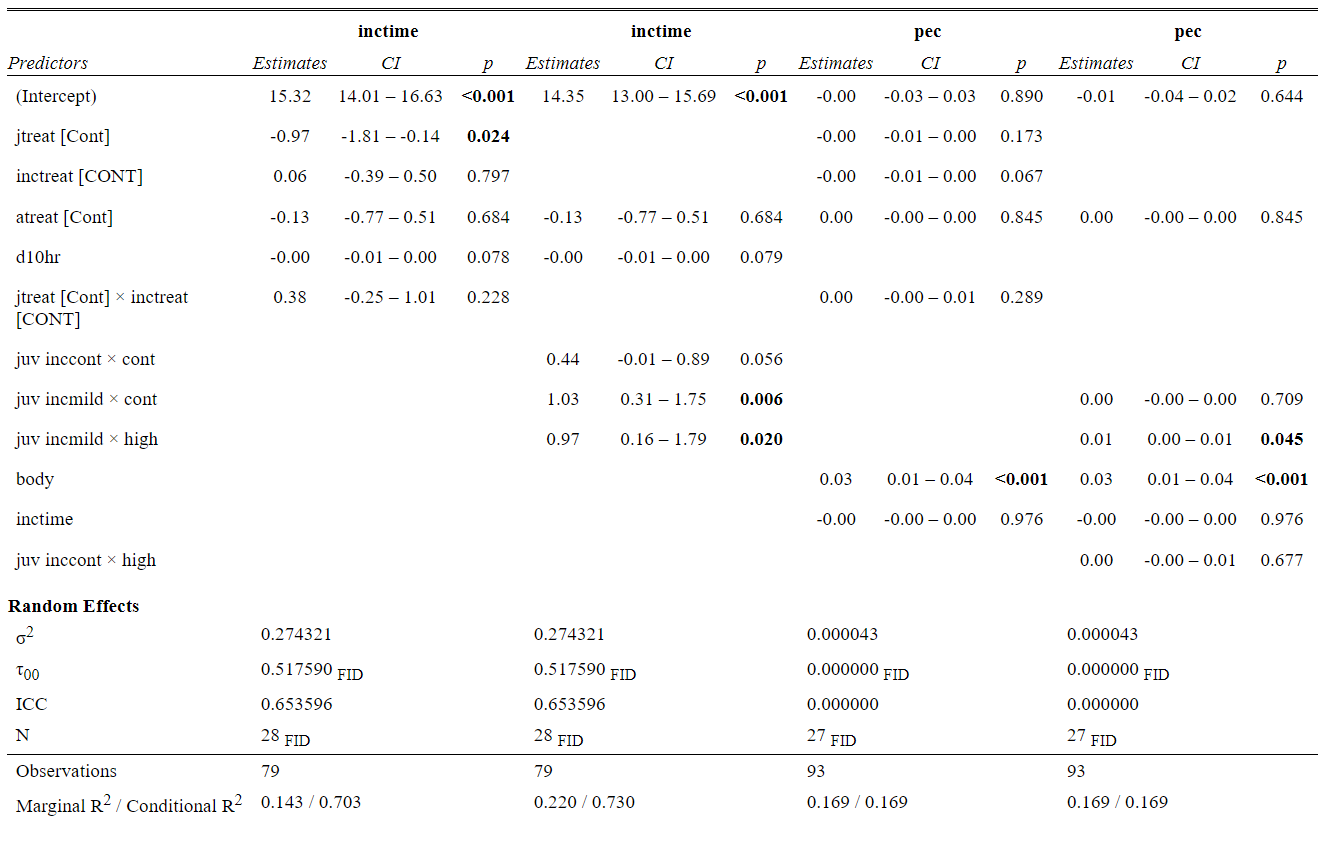
**

**Supporting Table S3. The statistical output of *LMMs* used to analyze the effect of maternal and incubation treatments on incubation duration (days) (“inctime”), and pectoralis mass of hatchlings (g) (“pec”). Two statistical models were used for analysis of each dependent variable, the first was an *LMM* with juvenile and incubation treatment as fixed factors, and a juvenile*incubation treatment interaction term. The second model was for post-hoc analysis and was done using a *LMM* which included a categorical treatment variable representing the four combinations of maternal juvenile and incubation treatment.**

**Supporting table S4**

**
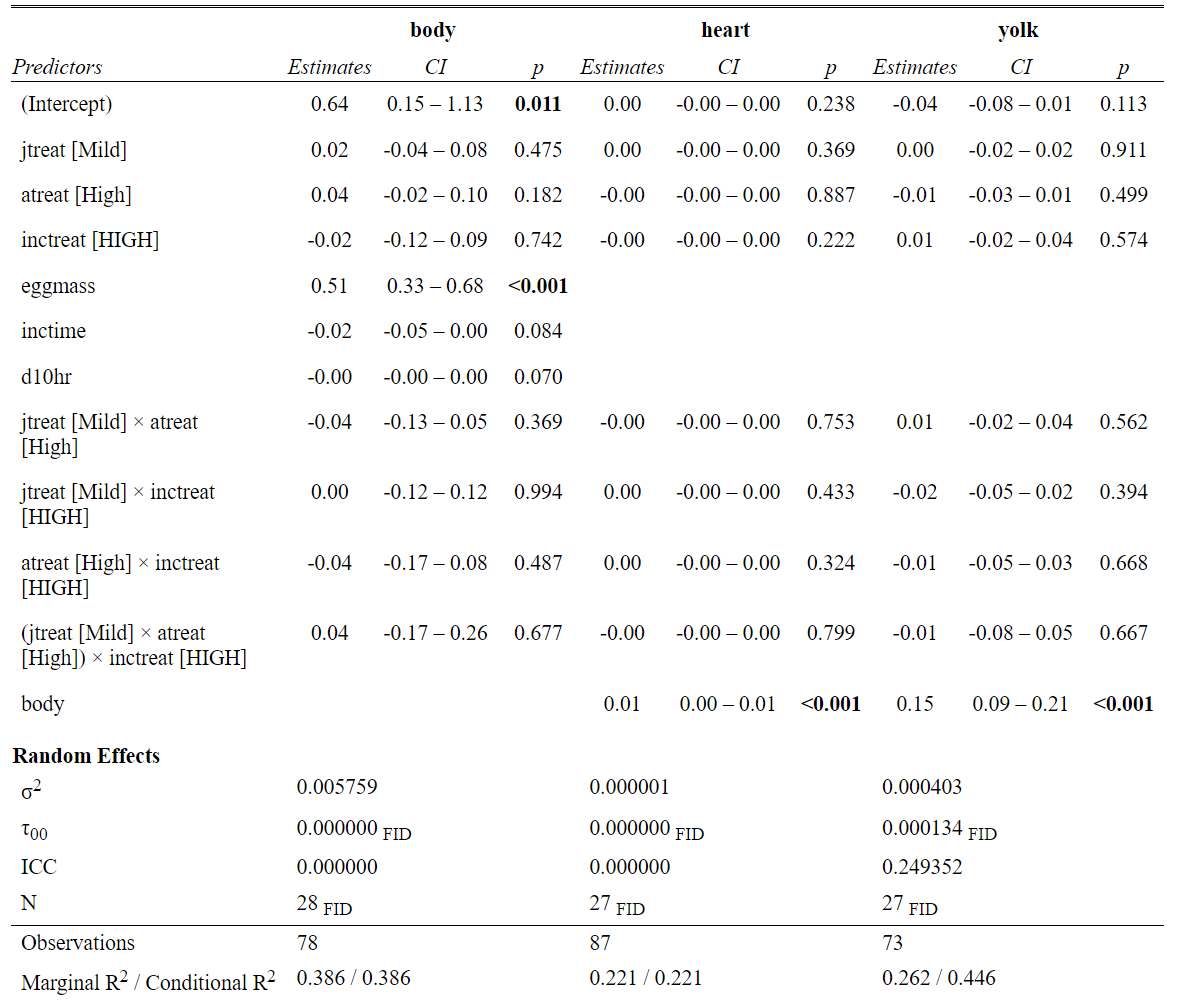
Supporting Table S4. The statistical output of *LMMs* used to analyze the effect of maternal and incubation treatment on hatchling total body mass (g)(“body”), heart mass (g)(“heart”), and residual yolk mass (g)(“yolk”).**

**Supporting table S5**

**
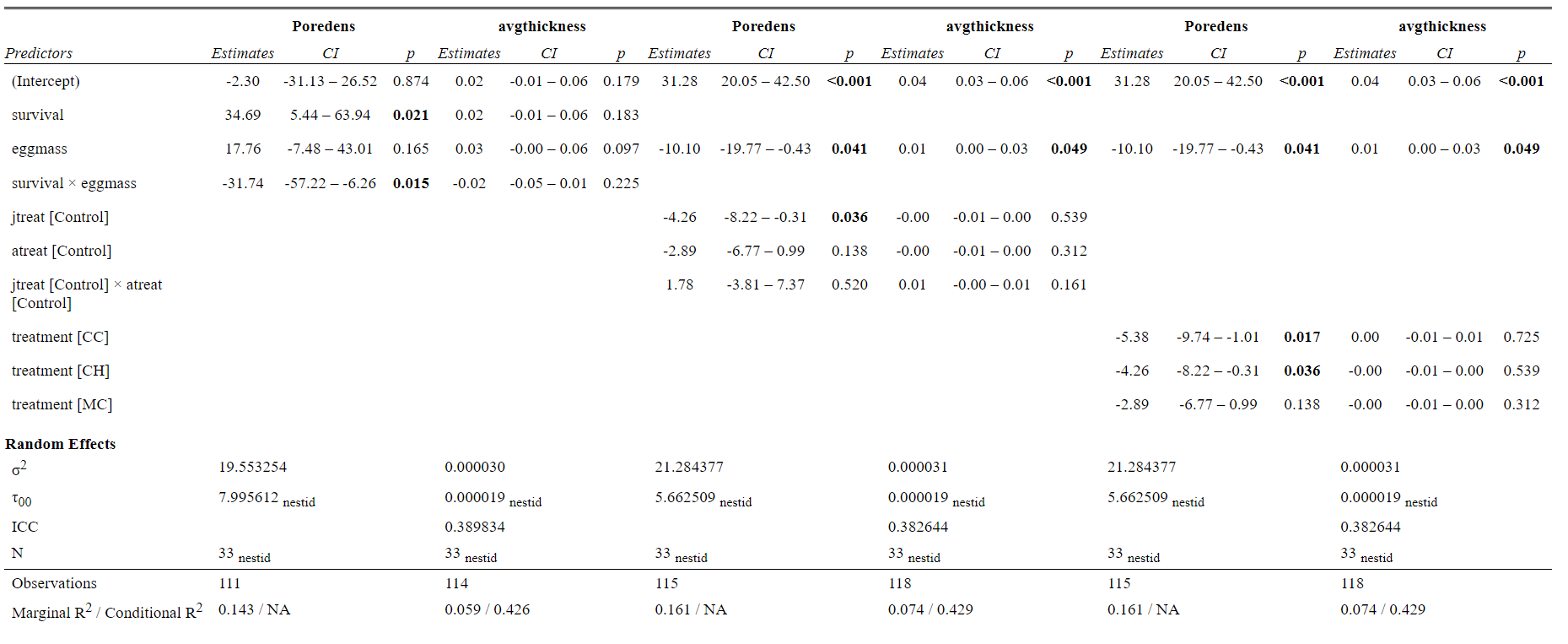
**

**Supporting Table S5. The statistical output of *LMMs* used to analyze the effect of eggshell pore density (pores/cm^2^) (“Poredens”) and average thickness (mm) (“avgthickness”) on embryo survival across all samples, as well as the effects of maternal and incubation treatments on eggshell pore density and thickness. For analysis of the effects of maternal and incubation treatments on eggshell pore density and thickness, two statistical models were used for each dependent variable. The first models were *LMMs* with maternal juvenile and adult treatment as fixed factors, and a juvenile*adult treatment interaction term. The second models were used for post-hoc analysis and were done using a *LMM*s which included a categorical treatment variable representing the four combinations of maternal juvenile and adult treatment.**

**Supporting table S6**


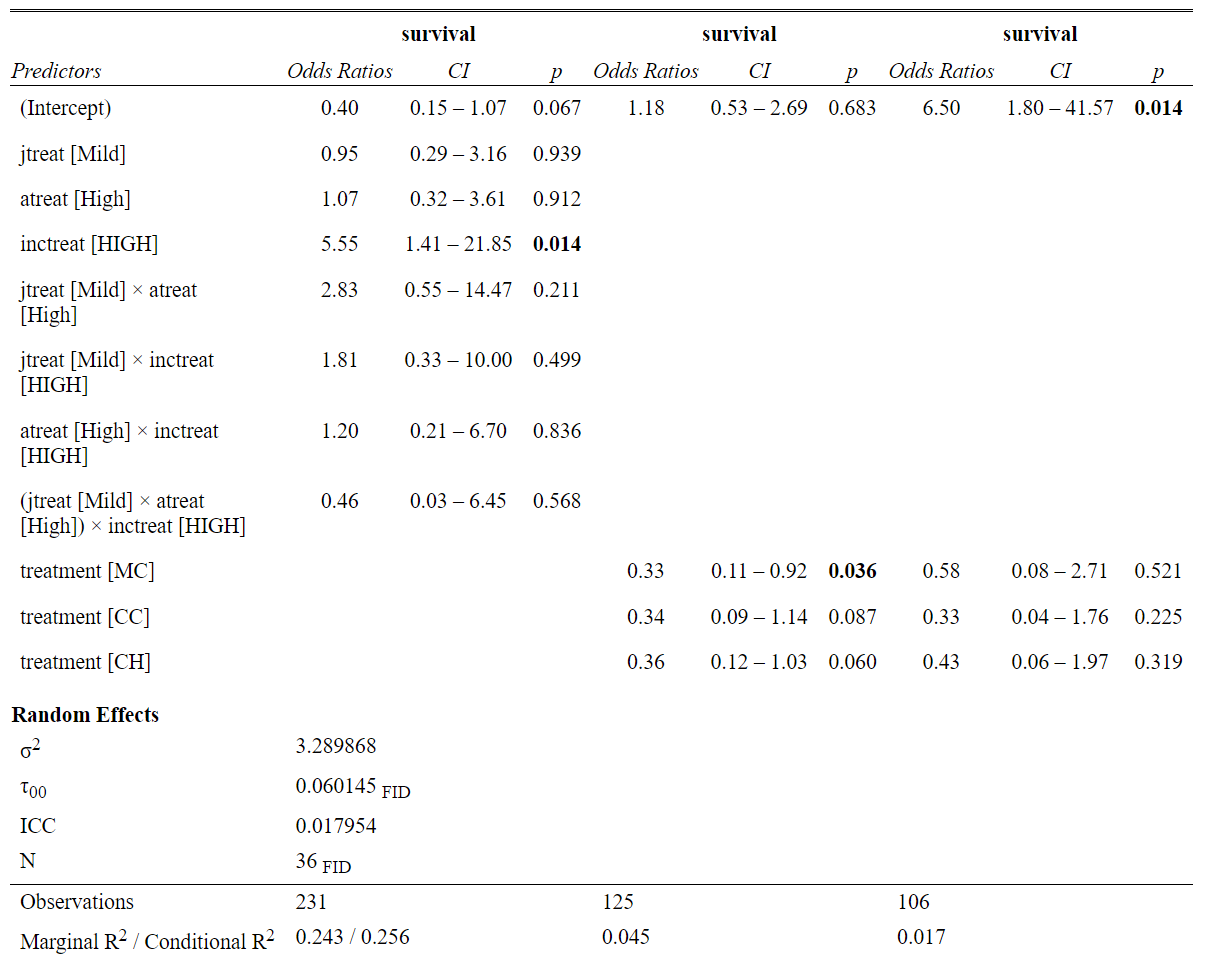


Survival

(control incubator)

Survival

(high incubator)

Survival

(both incubators)

**Supporting Table S6. The statistical output of *GLMs* used to analyze the effect of maternal and incubation treatments on embryo survival/hatch success. The first was a *GLMM* which included juvenile, adult, and incubation treatment as fixed effects, as well as the two- and three-way interaction terms. Two further *GLM*s were used for post-hoc analysis where the data was subset by incubation treatment (control or high) and included the maternal juvenile*adult categorical treatment variable as a fixed factor.**
